# Conflict Detection and Logical Complexity

**DOI:** 10.5334/pb.448

**Published:** 2018-11-16

**Authors:** Janie Brisson, Walter Schaeken, Henry Markovits, Wim De Neys

**Affiliations:** 1 Université du Québec à Montréal, CA; 2 University of Leuven, BE; 3 Paris Descartes University, Sorbonne Paris Cité, UMR 8240 LaPsyDÉ, FR; 4 CNRS, UMR 8240 LaPsyDÉ, FR

**Keywords:** Logical intuition, Conflict detection, Logical complexity

## Abstract

Empirical evidence for the capacity to detect conflict between biased reasoning and normative principles has led to the proposal that reasoners have an intuitive grasp of some basic logical principles. In two studies, we investigate the boundary conditions of these logical intuitions by manipulating the logical complexity of problems where logical validity and conclusion believability conflict or not. Results pointed to evidence for successful conflict detection on the basic Modus Ponens (MP) inference, but also showed evidence for such a phenomenon on the more complex Modus Tollens (MT) inference. This suggests that both the MP and the MT inferences are simple enough for reasoners to have an intuitive grasp of their logical structure. The boundaries of logical intuition might thus reside in problems of greater complexity than these inferences. We also observed that on the invalid Affirmation of the Consequent (AC) and Denial of the Antecedent (DA) inferences, participants showed higher accuracy on the inference that was expected to be more complex (DA), and no evidence for successful conflict detection was found on these forms. Implications for the logical intuition framework are discussed.

## Introduction

The biased nature of human inferential processes has been extensively demonstrated in decades of research on reasoning and decision making. Educated adults often violate the elementary principles of logic, probability or mathematics and favor fast and intuitive rules-of-thumb, called heuristics, to more deliberative thinking (Gilovich, Griffin & Kahneman, 2002). While an intuitive response can be congruent with the normative one, it sometimes conflicts with basic normative principles. A striking example of such a situation is the bat-and-ball problem (Frederick, 2005):

“A bat and a ball together cost $1.10. The bat costs $1 more than the ball. How much does the ball cost?”

Obviously, the correct answer is that the ball costs 5 cents and the bat costs $1.05 for a total of $1.10, but an intuitive answer “The ball costs 10 cents” is given by a vast majority of educated university students (Bourgeois-Gironde & Van der Henst, 2009). Of course, anyone who is familiar with the most basic principles of algebra should be able to come up with the right answer. So why do so many educated adults miss the goal? One answer would be that the parsing of $1.10 in $1 and 10 cents comes to mind so naturally that the intuitive “10 cents” answer becomes irresistible to many people (Kahneman, 2011).

However, it is unlikely that these biased people have no access to the normative response whatsoever. One question that arises from this is that when reasoners give an intuitive response that conflicts with a normative principle, are they perhaps aware of this conflict? Numerous studies have pursued this question by examining the detection of conflict in reasoning (e.g., De Neys & Glumicic, 2008; Pennycook, Fugelsang, & Koehler, 2015; Thompson & Johnson, 2014). The key manipulation of these studies is to present participants with problems for which the intuitive response conflicts with the normative one (like the above bat-and-ball problem) and a control version where both responses are the same. A no-conflict version of this problem could be:

“A bat and a ball together cost $1.10. The bat costs $1. How much does the ball cost?”

In this case, both mathematics and the intuitive parsing of $1.10 in $1 and 10 cents would lead to the same “10 cents” response.

Many studies have indicated that reasoners seem to process the conflict problems for which they gave the heuristic response differently than the no-conflict ones. For example, reasoners who answer intuitively to conflict problems need more time (Bonner & Newell, 2010; De Neys & Glumicic, 2008; Pennycook, Trippas, Handley, & Thompson, 2014; Villejoubert, 2009; Stupple, Ball, Evans, & Kamal-Smith, 2011), are less confident about their response (Bago & De Neys, 2017; De Neys, Cromheeke, & Osman, 2011; Gangemi, Bourgeois-Gironde, & Mancini, 2015; Johnson, Tubau, & De Neys, 2016; Thompson & Johnson, 2014) and show increased activation of brain areas assumed to mediate conflict and error monitoring (De Neys, Vartanian, & Goel, 2008; Simon, Lubin, Houdé, & De Neys, 2015) compared to when they give the normative answer to the no-conflict ones. These studies thus provide basic evidence for the presence of conflict detection in biased reasoners. That is, even when reasoners fall for an erroneous, intuitively cued response, they seem to show sensitivity to the fact that it is logically inappropriate.

This literature has led to the proposal that reasoners have an intuitive grasp of some basic logical principles (De Neys, 2012, 2014). The basic idea of this proposal is that when reasoners give a biased response to a logical problem, they can intuitively detect that something is wrong with their answer, but subsequently fail to override the prepotent biased response. Some form of comprehension of the logical principle at stake would thus be implied in the intuitive detection stage.

### Evidence for logical intuition: the “logical bias”

Many studies provide more general empirical support for this proposal. For example, some scholars have observed what we could call a “logical bias”. This bias is related to the well-known belief bias, that is the tendency to judge the validity of an argument based on the accordance between its conclusion and one’s beliefs rather than its logical structure. This heuristic makes people more prone to endorse an invalid argument when its conclusion is believable and reject a valid one when its conclusion contradicts one’s beliefs. However, recent studies have explored the possibility of a reversed phenomenon, that is, the possibility that the logical validity of an argument may bias judgments of believability. These studies presented participants with inferences where logic and belief were in accordance or in conflict and instructed them to evaluate their conclusions’ believability (Handley & Trippas, 2015; Trippas, Thompson, & Handley, 2017) or likability and brightness (Trippas, Handley, Verde, & Morsanyi, 2016) in a short period of time. They found that people needed more time and were more prone to errors when the logical validity of the problems conflicted with the conclusion’s believability. These studies thus provide empirical evidence for the presence of a “logic bias”. If logical processing would necessarily require slow and deliberate processing, then it should obviously not interfere with the evaluation of intuitive beliefs. These findings consequently lend credence to the proposal that people have an intuitive grasp of basic logical principles.

### Additional supportive finding: The two-response paradigm

Additional supportive studies of the logical intuition hypothesis use a two-response paradigm where participants are presented with reasoning problems and are first asked to give the first, intuitive response that comes to mind. To make sure that the initial response is truly intuitive in nature participants are given a short deadline or are imposed a cognitive load task (Bago & De Neys, 2017; Newman, Gibb, & Thompson, 2017). They are then given as much time as they need to give their final answer. These studies found that even in the fast and challenging conditions, participants generated correct logical responses and accepted valid inferences more often than the invalid ones, thus suggesting that a logical response can come from fast and intuitive processes.

### Critiques and boundary conditions of logical intuition

However, some critiques have been raised against studies on conflict detection and the logical intuition proposal. Some argue that the results supporting conflict detection could be due to a confound between the manipulation of conflict and the specific content or format of the problems (Aczel, Szollosi, & Bago, 2016; Klauer & Singmann, 2013; Singmann, Klauer, & Kellen, 2014). Others claim that incorrect responses to conflict problems, rather than being due to a failure to override the intuitive response, might arise earlier in the reasoning process and need to be attributed to an inaccurate comprehension of the problem at hand (Mata, Ferreira, Voss, & Kollei, 2017; Mata, Schubert, & Ferreira, 2014). Others address the extent of the proposal, arguing that the capacity to detect conflict might be limited to tasks where the contrast between the intuitive and the normative answers is amplified (Pennycook, Fugelsang, & Koehler, 2012) or when the underlying principle is simple (Travers, Rolison, & Feeney, 2016). These latter critiques lead to a key open question, that will be the major focus of the current studies, namely to define the boundary conditions of logical intuitions. Can we assume that reasoners have a logical intuition about each and every problem? Our stance on this question is rather that, except for highly trained logicians, logical intuitions arise only for simple problems to which people have been exposed frequently enough to develop an intuitive sense of their structure (De Neys, 2012, 2014). We thus posit the main hypothesis that, when presented with logical problems that contain a more complex structure, reasoners will be less likely to show conflict detection since it will be less likely that a logical intuition will arise from the task.

For completeness, it should be noted that the logical intuition concept plays a key role in several (related) recent dual process models. For example, as the basis of a so-called hybrid dual-process account (De Neys, 2018), as a component of the three-stage model (Pennycook, 2018), or occurring alongside belief-based thinking in a parallel-processing model (Trippas & Handley, 2018). Rather than being a test for theory, the following studies are an empirical enquiry regarding the scope of logical intuitions, and can thus be a useful contribution to all of these models.

In continuity with previous studies on conflict detection in logical reasoning (Bago & De Neys, 2017; De Neys & Franssens, 2009; De Neys, Moyens, & Vansteenwegen, 2010) we used simple categorical syllogisms that were first introduced by Markovits and Nantel (1989) and popularized through the work of Stanovich and West (1998, 2000; West, Toplak, & Stanovich, 2008) and others. The first syllogism starts with the premises “All As are Bs; All Cs are As” and leads to the valid conclusion “All Cs are Bs”. For example:

“All dogs have legs
Labradors are dogs
Therefore, Labradors have legs”

The second starts with the premises “All As are Bs; All Cs are Bs” and lead to the invalid conclusion “All Cs are a As”. Such an inference could be:

“All dogs have legs
Labradors have legs
Therefore, Labradors are dogs”

This latter conclusion is invalid since many animals besides dogs can have legs. The conclusion thus follows possibly, but not necessarily, from the premises.

These syllogisms are categorical versions of two conditional inferences. The first is equivalent to the valid Modus Ponens or MP (If P then Q, P is true, therefore Q is true) and the second is equivalent to the invalid Affirmation of the Consequent or AC (If P then Q, Q is true, therefore P is true). Previous developmental research has suggested that categorical syllogisms are simpler to process than their propositional counterpart (Markovits, 2017). We thus started with these items as a lower bound in our enquiry for evidence of logical intuitions. Note that we will refer to these syllogisms as MP and AC, respectively.

One way to directly look for an upper boundary conditions of logical intuitions would be to increase these problems’ complexity. These MP and AC inferences are indeed simple and one element of their simplicity is that their premises are always affirmed. One way to increase their complexity is to include negations in their premises and conclusion, as it is well established that the presence of a negation adds a cognitive burden to the reasoning process (Schaeken & Schroyens, 2000; Schroyens, Schaeken, Fias & d’Ydewalle, 2000; Schroyens, Schaeken & d’Ydewalle, 2001). Adding a negation to our previous MP syllogism could result in:

“All dogs have legs
Cats are not dogs
Therefore, cats don’t have legs”

The syllogism would then become invalid and be equivalent to the Denial of the Antecedent conditional inference (If P then Q, P is false, therefore Q is false). Moreover, adding a negation to the AC syllogism could result in:

“All dogs have legs
Cats don’t have legs
Therefore, cats are not dogs”

Which would equate to the valid Modus Tollens (If P then Q, Q is false, therefore P is false). Note that we will refer to these two syllogisms as DA and MT, respectively.

We will thus use these four inference forms to manipulate logical complexity in order to test for the boundary conditions of logical intuition, MP and AC being the simple inferences and MT and DA the more complex ones. Note that, while conflict detection on propositional versions of MP and MT has been previously observed (Trippas et al., 2017), to our knowledge, our studies are the first to manipulate complexity with these categorical items and with these four inferences forms.

We hypothesized that for more complex inferences, it will be less likely that people will have an intuitive grasp of their logical structure. Consequently, previously observed conflict detection effects (e.g., increased response latencies for conflict problems) should be less likely with the complex inferences than with the simpler ones.

## Pretest

To test our hypothesis, we first constructed four sets of 16 categorical syllogisms for a pretest. Within each set, conclusion believability was in conflict with validity for half of the problems (i.e., conflict problems; two unbelievable MP, two unbelievable MT, two believable AC and two believable DA). For the other half, believability was consistent with validity (i.e., no-conflict problems; two believable MP, two believable MT, two unbelievable AC and two unbelievable DA). In each problem set, two additional conflict and two no-conflict syllogisms were added, for a total of 20 syllogisms in each set. This addition was done in order to be able to afterwards select (for the main study) a final set of 64 conclusions shown to be maximally believable or unbelievable in the pretest.

To minimize the possibility that content related variability would affect our results, we crossed the item content with the conflict status and logical form complexity across the four sets. That is, with the same major premise, we switched the order of the minor premise and the conclusion to manipulate problem validity, thus turning MP problems into an AC form and MT problems into a DA form, and vice versa. To make sure that the minor premise was always believable, we also changed the minor term of the unbelievable conclusions. Consequently, across the four blocks, the same major premise was used to construct a different type of problem. A major premise that was used in one block to construct a simple conflict problem would be used to construct a complex no-conflict problem in another block, etc. This is a first step to minimize the possibility that simple item content differences bias the effect of problem complexity or conflict.

The conclusion believability classification in our items sets was based on previously published classifications and our own intuitions. To validate the classification and avoid confounds, we ran an extensive believability rating pretest for the 80 conclusions in our item sets.

Twelve participants took part in the pretest (3 females, 9 males, Mean age = 38 years, 4 months). We asked them to rate the believability of each conclusion on a scale of 0 to 10 (0 being totally unbelievable and 10 being totally believable). Conclusions from the item sets were presented in four different blocks, each block consisting of the 20 conclusions of one item set. Questions within each set were presented to participants in a randomized fixed order.

We first calculated the mean believability ratings for each of the 80 conclusions. We discarded 16 conclusions with moderate ratings, so that each conclusion in our item sets would be maximally believable (close to 10) or unbelievable (close to 0) with as little variance as possible.

To check whether the average believability of the selected material did not systematically differ, we performed a 2 (Conflict: conflict, no-conflict) × 2 (Complexity: easy, hard) within-subject ANOVA on believability ratings. Average ratings for conflict (M = 5.12, SE = 1.02) and no-conflict problems (M = 5.11, SE = 1.05) and simple (M = 5.292, SE = 1.1) and complex problems (M = 4.93, SE = 0.97) were very close. The ANOVA showed no significant effect of Conflict, F(1, 15) < 1, nor Complexity, *F*(1, 15) = 2.532, *p* = 0.13, and no significant interaction between Conflict and Complexity *F*(1, 15) = 2.409, *p* = 0.14. We can thus minimize the possibility that effects resulting from our manipulation of conflict and complexity on conflict detection will be attributable to differences in conclusions believability. Appendix A gives a complete overview of the 16 selected problems in the four item sets and Appendix B gives a overview of the mean believability ratings for each of their conclusions.

## Study 1

### Method

**Participants.** A total of 95 participants (53 males, 42 females, Mean age = 32 years, 1 month) were recruited via the online Crowdflower platform and received $0.30 for their participation. Only native English speakers from the USA or Canada were allowed to participate in the study. A total of 31.6% of participants reported high school as highest completed educational level, 2.1% reported not having a high school degree and 66.3% reported having a post-secondary education degree. Note that previous studies have shown that both laboratory based (e.g., De Neys & Glumicic, 2008) and online settings (e.g., Frey, Johnson, & De Neys, 2017) give similar latency results. This online administration is thus in continuity with the relevant literature on conflict detection.

**Material and procedure.** The four item sets selected in the pretest were used in this experiment. Participants were randomly divided into four groups to which one item set was assigned. Items were presented to them in a randomized order. Hence, each participant solved a total of 16 problems. Half of these were conflict problems (i.e., two unbelievable MP, two unbelievable MT, two believable AC and two believable DA) and half were no-conflict problems (i.e., two believable MP, two believable MT, two unbelievable AC and two unbelievable DA). No time limit was imposed. All participants were first given the following instructions.

“In this experiment, you will need to solve a number of reasoning problems. In each problem you are going to get two premises, which you have to assume being true. Below the premises you will see a conclusion. We ask you to determine whether the conclusion follows logically from the premises or not. You have to assume that the premises are all true. This is very important.

Below you can see an example of the problems:

- Premise 1: All dogs have four legs
- Premise 2: Puppies are dogs
- Conclusion: Puppies have four legs
- Does the conclusion follow logically?

- Yes
- No

Once you have made up your mind we ask you to immediately click on the corresponding answer option. Then you have to click on the red “Next” button to advance to the next problem. Press “Next” if you are ready to start the practice session!”

Participants then received a practice session (not analyzed) of one additional item. They then proceeded to answer the 16 reasoning problems. Half of participants were randomly assigned to solve 8 easy problems first followed by 8 hard problems and half were randomly assigned to a reversed order of complexity. They were then all asked to provide basic demographic information.

### Results and discussion

A preliminary data check showed that 4 participants took an unusually short time to read the instructions page (less than 2 seconds, whereas average reading time was 78s, SD = 314s). We therefore decided to discard the data of these four participants from further analysis.

**Manipulation check: Accuracy findings.** We first wanted to verify whether our complexity manipulation was successful. Are the complex inferences really harder to solve than the simple ones? We therefore first looked at accuracy. Table 1 shows the results. Overall, participants performed better on the easy (M = 65.1%, SD = 21%) than on the hard problems (M = 59.6%, SD = 18.1%), *t*(90) = 2.81, *p* < 0.01. However, examination of Table 1 indicates that this did not hold for all problem types. On the valid MP/MT problems, we did observe the expected pattern with higher accuracies throughout on the easy MP problems than on the complex MT problems, (M = 78.6%, SD = 23.4%; M = 63.7%, SD = 31.5%, respectively), *t*(90) = 4.57, p < 0.001. However, the difference between the invalid AC and DA problems was not significant (M = 51.7%, SD = 32.2%; M = 55.5%, SD = 34.1%, respectively), *t*(90) = –1.41, *p* = 0.16.

**Table 1:** Percentage of logically correct responses on overall, conflict and no-conflict problems for Study 1, Study 2, and pooled data (standard deviations in parentheses).

	Logical form	Overall	Conflict	No-conflict

Study 1	MP	78.6 (23.4)	63.2 (40.7)	94 (16.4)
	MT	63.7 (31.5)	54.9 (40.2)	70.3 (38.7)
	AC	51.7 (32.2)	33. (41)	72.5 (37.5)
	DA	55.5 (34.1)	42.9 (43.8)	68.1 (40.5)
Study 2	MP	81.1 (23.7)	63.3 (45.3)	98.9 (7.3)
	MT	68.4 (32.8)	60.6 (44.5)	88.8 (24.5)
	AC	73.9 (29.3)	59. (46.4)	76.1 (34.2)
	DA	76.1 (30.4)	65.4 (42.8)	86.7 (27.6)
Pooled data	MP	79.9 (23.5)	63.2 (43)	96.5 (12.8)
	MT	66.1 (32.2)	57.8 (42.4)	79.7 (33.5)
	AC	63 (32.7)	46.2 (45.6)	74.3 (35.8)
	DA	66 (33.8)	54.3 (44.6)	77.6 (35.7)

Our manipulation of complexity was thus in line with expectations for MP and MT but not for AC and DA. Surprisingly, response accuracy did not indicate that the DA inference was harder than the AC inference. Obviously, the accuracy findings complicate a test of our main conflict detection hypothesis for the AC/DA problems. Since problem complexity was a key manipulation for our hypothesis, further analysis on conflict detection will be done for valid MP/MT and invalid AC/DA inferences separately.

#### Conflict detection analysis

Preliminary analysis of latencies showed positive skewness for failed conflict MP (skewness = 3.05, SE = 0.51) and AC (skewness = 3.01, SE = 0.51) as well as succeeded no conflict MT (skewness = 2.20, SE = 0.51). All latencies were thus log-transformed before further analysis. See Table 2 for an overview of the raw latency findings.

**Table 2:** Response latency (in seconds) for incorrect conflict and correct no-conflict problems (standard deviations in parentheses).

	Logical form	Conflict	Accuracy	Response time	Conflict detection effect*

Study 1	MP	Conflict	Incorrect	10.09 (9.88)	1.83
		No-Conflict	Correct	8.26 (6.79)	
	MT	Conflict	Incorrect	11.78 (11.82)	3.57
		No-Conflict	Correct	8.27 (6.52)	
	AC	Conflict	Incorrect	7.5 (6.1)	–1.65
		No-Conflict	Correct	9.15 (5.21)	
	DA	Conflict	Incorrect	10.24 (7.29)	–1.03
		No-Conflict	Correct	11.26 (6.9)	
Study 2	MP	Conflict	Incorrect	6.29 (6.58)	2.58
		No-Conflict	Correct	3.71 (2.42)	
	MT	Conflict	Incorrect	7.7 (10.73)	2.78
		No-Conflict	Correct	4.89 (2.43)	
	AC	Conflict	Incorrect	3.46 (3.67)	–0.98
		No-Conflict	Correct	4.44 (3.45)	
	DA	Conflict	Incorrect	6.61 (8.14)	–1
		No-Conflict	Correct	6.61 (7.56)	
Pooled	MP	Conflict	Incorrect	8.19 (8.53)	2.21
		No-Conflict	Correct	5.98 (5.55)	
	MT	Conflict	Incorrect	9.74 (11.38)	3.16
		No-Conflict	Correct	6.58 (5.16)	
	AC	Conflict	Incorrect	5.64 (5.48)	–1.34
		No-Conflict	Correct	6.98 (5.04)	
	DA	Conflict	Incorrect	8.57 (7.84)	–1.01
		No-Conflict	Correct	9.58 (7.38)	

*Note.* *Incorrect conflict minus correct no-conflict trials latency difference. More positive values indicate stronger detection effect.

***Valid inferences.*** Consistent with previous conflict detection studies (e.g., De Neys & Glumicic, 2008; De Neys et al., 2010; Frey, Johnson & De Neys, 2017), to test for conflict detection, we analyzed response latency on MP and MT for participants who failed the conflict items and succeeded the no-conflict items. We then performed a 2 (Conflict: incorrect conflict, correct no-conflict) × 2 (Complexity: MP, MT) within-subject ANOVA on response latency. First, a main effect of Conflict showed that when participants failed the conflict problems, they took more time (M = 10.93, SD = 10.85) than when they succeeded the no-conflict ones (M = 8.26, SD = 6.65), *F*(1, 27) = 5.073, *p* < 0.05, *partial eta^2^* = 0.16. We found no significant effect of Complexity, *F*(1, 27) < 1, and, critically, no significant interaction between Conflict and Complexity, *F*(1, 27) < 1.

First, these results suggest that reasoners are sensitive to the conflict between their biased response and the logical one, which is in line with our general hypothesis on conflict detection. However, the absence of interaction between Conflict and Complexity suggests that, contrary to our predictions, our manipulation of complexity had no significant impact on conflict detection. Indeed, as Table 2 indicates, if anything there was a trend towards a slightly stronger conflict detection effect on the harder MT than on the easier MP problem.

***Invalid inferences.*** We then ran the same analysis on the invalid problems. A 2 (Conflict: incorrect conflict, correct no-conflict) × 2 (Complexity: AC, DA) within-subject ANOVA on response latency. A main effect of Complexity showed that participants took more time to solve the DA (M = 10.75, SD = 7.09) than the AC (M = 8.32, SD = 5.65) inference, *F*(1, 33) = 11.599, *p* < 0.01, *partial eta^2^* = 0.26. Moreover, a marginally significant main effect of Conflict suggested that, contrary to our predictions, participants took more time to solve the correct no-conflict problems (M = 10.206, SD = 6.05) than the incorrect conflict ones (M = 8.87, SD = 6.69), *F*(1, 33) = 3.358, *p* = 0.076, *partial eta^2^* = 0.09. No significant interaction between Conflict and Complexity was found, *F*(1, 33) = 1.094, *p* = 0.3. These results thus show that we did not replicate the evidence for conflict detection on the AC inference and that we did not find such evidence for the DA inference.

These unexpected results on accuracy and conflict detection with the invalid inferences are puzzling. Study 2 was run to test the robustness of these effects. Moreover, one possible limitation of Study 1 is that we did not control for differences in premises reading time. Stimulus presentation was thus slightly modified in Study 2 in order to provide a finer measurement of reasoning latencies.

## Study 2

### Method

**Participants.** A total of 96 participants (50 males, 45 females, Mean age = 34 years, 10 months, one participant failed to indicate demographic information) were recruited via the online Prolific Academic platform and received $0.68 for their participation. Only native English speakers from the United States, Canada, United Kingdom, New Zealand and Australia were allowed to participate in the study. A total of 35.4% of participants reported high school as highest completed educational level and 63.6% reported having a post-secondary education degree.

**Material and procedure.** We used the exact same material and procedure as in Study 1, except that we presented the premises and conclusion serially. That is, participants first saw the major and minor premises and were instructed to click “next” once they finished reading them. The conclusion and question were then added to the premises, which completed the reasoning problem. Response latencies were calculated from then. We reasoned that removing premises’ reading times from the response latencies might result in more accurate measurement of the actual reasoning time.

### Results and discussion

As in Study 1, preliminary data checking indicated that 2 participants showed a deviant short time to read the instructions page (less than 2.1 seconds; with an average reading time of 50s, SD = 64s). Data from these two participants was discarded from further analysis.

**Manipulation check: accuracy findings.** We first looked at our complexity manipulation. Table 1 shows the results. As in Study 1, participants performed better overall on the easy (M = 77.5%, SD = 22.6%) than on the hard problems (M = 72.2%, SD = 21.9%), *t*(93) = 2.75, *p* < 0.01. As expected, we observed higher accuracies on MP (M = 81.2%, SD = 23.6%) than on the MT problems, (M = 68.4%, SD = 32.8%), *t*(93) = 3.88, *p* < 0.001. Once again the difference between the AC (M = 73.9%, SD = 29.3%) and DA (M = 76.1%, SD = 30.4%) inferences was not significant, *t*(93) = – 0.79, *p* < 0.43. These results thus replicate what was found in Study 1. Our manipulation of complexity being supported only for MP/MT, conflict detection will again be analyzed separately for valid and invalid forms.

#### Conflict detection analysis

As in study 1, preliminary analysis of latencies showed positive skewness for some variables, namely failed conflict MP (skewness = 2.26, SE = 0.55), MT (skewness = 3.07, SE = 0.55), AC (skewness = 3.15, SE = 0.55) and DA (skewness = 2.46, SE = 0.55) as well as succeeded no conflict DA (skewness = 3.55, SE = 0.55). Further analyses were thus conducted on log-transformed data (see Table 2 for raw latencies).

***Valid inferences.*** We then analyzed response latency on MP and MT for participants who failed the conflict items and succeeded the no-conflict one. As in Study 1, we performed a 2 (Conflict: incorrect conflict, correct no-conflict) × 2 (Complexity: MP, MT) within-subject ANOVA on log-transformed response latencies. A marginally significant main effect of Conflict suggested that when participants failed the conflict problems, they took more time (M = 6.99, SE = 1.2) than when they succeeded the no-conflict ones (M = 4.3 SE = 0.34), *F*(1, 27) = 3.933, *p* = 0.058, *partial eta^2^* = 0.13. No significant effect of Complexity, *F*(1, 27) = 1.794, *p* = 0.19, and no significant interaction between Conflict and Complexity were found, *F*(1, 27) = 1.305, *p* = 0.26. Although the results were marginally significant, this study suggests a replication of the conflict detection findings for both the MP and MT inferences, with our manipulation of complexity having no significant impact on the effect.

***Invalid inferences.*** We then ran the same analysis on the invalid problems. A 2 (Conflict: incorrect conflict, correct no-conflict) × 2 (Complexity: AC, DA) within-subject ANOVA on response latency showed the same pattern as observed in Study 1. A main effect of Complexity showed that participants took more time to solve the DA (M = 7.11, SE = 0.77) than the AC (M = 4, SE = 1.07) inference, *F*(1, 28) = 27.296, *p* < 0.001, *partial eta^2^* = 0.49. There was a marginal significant main effect of Conflict, *F*(1, 28) = 3.682, *p* = 0.07 that pointed to *longer* latencies on the no-conflict problems. Finally, Conflict and Complexity did not interact, *F*(1, 28) < 1.

The purpose of this study was to validate the unexpected findings of Study 1, that is, evidence for conflict detection on the valid MP and MT inference with no difference across complexity and no evidence for conflict detection for the invalid AC and DA forms. The results found here did replicate those findings, although conflict detection on the valid forms was marginally significant. Provided that both studies showed the same tendencies, we decided to pool the data obtained in both studies and apply the same analysis with increased power.

### Pooled conflict detection analysis

Regarding conflict detection on the valid inferences, a 2 (Conflict: incorrect conflict, correct no-conflict) × 2 (Complexity: MP, MT) within-subject ANOVA on response latency gave a significant main effect of Conflict, which showed again that when participants failed the conflict problems, they took more time (M = 8.97, SE = 0.97) than when they succeeded the no-conflict ones (M = 6.28, SE = 0.6), *F*(1, 55) = 8.79, *p* < 0.01, *partial eta^2^* = 0.14. We found no significant effect of Complexity, *F*(1, 55) = 1.713, *p* = 0.2, and no significant interaction between Conflict and Complexity, *F*(1, 55) < 1.

With respect to the invalid problems, a 2 (Conflict: incorrect conflict, correct no-conflict) × 2 (Complexity: AC, DA) within-subject ANOVA on response latency gave a main effect of Complexity, which showed that participants took more time to solve the DA (M = 9.08, SE = 0.71) than the AC (M = 6.31, SE = 0.55) inference, *F*(1, 62) = 36.029, *p* < 0.001, *partial eta^2^* = 0.37. There was also a main effect of Conflict, but in contrast with the conflict detection hypothesis, the effect was again reversed so that when participants failed the conflict problems, they took less time (M = 7.11, SE = 0.76) than when they succeeded the no-conflict ones (M = 8.28 SE = 0.65), *F*(1, 62) = 7.143, *p* < 0.05, *partial eta^2^* = 0.10. No significant interaction between Conflict and Complexity was found, *F*(1, 55) = 1.228, *p* = 0.27.

### Conflict and believability confound

As explained in the presentation of the material, our conflict problems were created by crossing problem believability with logical validity, so that both believable–invalid problems and unbelievable–valid problems were constructed. Given the unexpected accuracy findings, we analysed the valid and invalid problems separately. One possible limitation that arises from this is that our manipulation of conflict was collapsed with conclusion believability. That is, in the valid problems, the conflict versions are always unbelievable whereas no-conflict problems were believable. Similarly, for invalid problems the conflict versions were always believable whereas no-conflict problems were unbelievable. Hence, one alternative explanation for our conflict findings is simply that people take more time to evaluate unbelievable conclusions. This would lead to a “conflict detection” effect on valid problems and a reversed effect (i.e., longer latencies for no-conflict problems) on invalid problems – exactly the pattern we observed in our pooled analysis. One way to control for this potential confound is to test for an effect of conflict for problems that are matched on believability (i.e., valid unbelievable vs. invalid unbelievable and invalid believable vs. valid believable). Hence, conflict and no conflict problems differ in validity but not in believability. In other words, in this matched control analysis the “conflict” effects always contrast valid and invalid problems rather than validity and believability. If participants are sensitive to logical validity and not simply believability per se, they should still show longer latencies for conflict versions here. Therefore, in a control analysis on our pooled Study 1 and 2 data we also tested for the effect of this conflict factor on response latencies. Believability was entered as a separate factor in the design. This resulted in a 2 (Conflict: conflict, no-conflict) × 2 (Believability: believable, unbelievable) ANOVA on response latencies. A main effect of Believability showed that overall, participants took less time to solve the believable (M = 8.12, SE = 0.52) than the unbelievable problems (M = 10.1, SE = 078), *F*(1, 184) = 22.836, p < 0.001, *partial eta^2^* = 0.11. Critically, a main effect of Conflict, *F*(1, 184) = 12.238, *p* = 0.001, *partial eta^2^* = 0.06, showed that participants still took more time to solve the conflict, (M = 9.39, SE = 0.63) than the no conflict problems, (M = 8.83, SE = 0.72), even when the believability status was matched. No interaction between Conflict and Believability was found, *F*(1, 184) < 1. This establishes that our overall results are not driven by a believability confound but by the presence or absence of conflict between logical validity and believability per se.

#### Bayesian null-effect complexity test

With respect to the valid inferences, our pooled analysis corroborated the presence of conflict detection for both the MP and MT inferences without any effect of complexity. If anything, the effect tended to be more pronounced on the harder MT then on the easier MP inference. However, the critical conclusion with respect to a lack of complexity effect is based on a null finding. Even though we boosted power in a pooled analysis, the p-value significance testing approach presented here cannot quantify a degree of support for the null hypothesis. To address this issue, we relied on Bayesian hypothesis testing using Bayes factors (e.g., Masson, 2011; Wagenmakers, 2007). Using the JASP package (JASP Team, 2017), we ran a 2 (Conflict: incorrect conflict, correct no-conflict) × 2 (Complexity: MP, MT) Bayesian ANOVA on response latencies for the valid inferences with default priors (e.g., Cauchy prior width r = .707). This showed that the model with a main effect of Conflict received the most support against the Null model (BF10 = 1.52). Adding the interaction with Complexity to the model decreased the degree of support against the Null model (BF10 = 0.138). The model with a main effect of Conflict was thus preferred to the interaction model by a Bayes factor of 11.01. These data thus provide good evidence against the hypothesis that conflict detection is modulated by complexity on the MP and MT inferences (see Wetzels et al., 2011 for a classification of Bayes factors).

## General Discussion

In these studies, we aimed to test the boundary conditions of logical intuitions (De Neys, 2012). To this end, we manipulated logical complexity and expected that conflict detection would be smaller for inferences of greater complexity. However, some of our results were unexpected and contrary to predictions.

The most unclear and surprising results concerned the invalid AC and DA inferences. First, our accuracy results indicated that the DA was not harder than the AC inference. We don’t have a clear explanation for this finding, but these results were robust amongst both our studies and should clearly call for further investigation. One suggestion would be that the effect of negation on AC and DA inferences is less clear than for their valid counterparts, as previous studies have shown variable frequencies of endorsement for these inferences (see Evans, 1993 for a review).

We also failed to observe the expected conflict detection effect on the invalid AC form. Note that previous studies that reported successful conflict detection with simple AC and MP problems typically ran analyses in which performance over both inference types was collapsed (e.g., Bago & De Neys, 2017; De Neys et al., 2010; De Neys & Franssens, 2009). Hence, the present results suggest that these effects were primarily driven by the MP inference.

Our results on the valid MP and MT inferences did provide additional support for the general idea that biased reasoners can show sensitivity to the conflict between their heuristic response and logical principles. However, contrary to our predictions, we observed conflict detection for both the easy MP form and for the more difficult MT form. This leads to a number of interesting considerations. First, our basic manipulation of problem complexity was the presence/absence of negations. While there is empirical support for the additional cognitive challenge that negation provides (Schaeken & Schroyens, 2000; Schroyens et al., 2000, 2001), it might not be strong enough for us to observe differences in conflict detection. This possibility would be congruent with the results of Trippas et al. (2017), who indicated in their supplementary material that they found no significant difference in conflict detection between the propositional versions of MP and MT, but still reported smaller conflict detection effects for syllogisms of greater complexity than MT. Combined with these results, our study suggests that MT might be simple enough for people to develop an intuitive grasp of its structure. With hindsight one could argue here that even though MT might be harder than MP, the MT inference is still a basic form of argument refutation and similar patterns (like the *reductio ad absurdum* proof) are taught in school. Consequently, through such repeated exposure, MT might still be simple and frequent enough to allow for the development of logical MT intuitions. Second, although the effects were not significant, our conflict detection results indicated that conflict detection was even more pronounced on MT than MP. Provided that conflict can be detected both intuitively and deliberately, one speculative possibility is that MT’s slightly greater complexity triggered more deliberative reasoning processes, thus making reasoners more likely to detect conflict with these inferences. If this was the case, complexity and conflict detection could be linked by a reversed U-shape relation, thus making conflict detection optimal when problem complexity is intermediate and then dropping as complexity increases. Of course, further studies would be needed to investigate this possibility.

As explained above, our manipulation of conflict was collapsed with conclusion believability. While we extensively pretested our material and an additional control analysis showed that conflict effects were observed even when the believability status was matched, a potential believability confound can still be stressed as a possible limitation for our studies. Note, however, that this confound is inherent to the manipulation of belief with the categorical syllogisms used here. When crossing validity and believability the content of the categorical syllogisms needs to be altered so that a potential believability confound can never be ruled out completely. Future studies on the complexity question might thus adopt different types of problems, where the conflict manipulation allows a full counterbalancing of the content material (e.g., base-rate problems, e.g., Pennycook et al., 2015). Moreover, since the logical intuition proposal goes beyond mere reasoning with categorical syllogisms, testing a wider range of basic logical principles would not only be methodologically but also theoretically relevant to clearly delineate its extents and limits.

In sum, it is important to stress that the present results do clearly not entail that there are no boundary conditions for logical intuitions. Indeed, the logical intuition proposal is a post hoc theoretical inference posited to account for observed empirical data (De Neys, 2012). Further studies will thus be needed in order to explore these boundaries. This study, however, has informed this question by indicating that they presumably reside in problems of greater complexity than the MT inference.

## Additional Files

The additional files for this article can be found as follows:

- pb-58-1-448-s1.pdf
  **Appendix A** Item sets. DOI: https://doi.org/10.5334/pb.448.s1
- pb-58-1-448-s1.pdf
  **Appendix B** Mean conclusion believability (on a scale of 0 to 10) for each conclusion. DOI: https://doi.org/10.5334/pb.448.s1
